# Statin use is associated with lower disease severity in COVID-19 infection

**DOI:** 10.1038/s41598-020-74492-0

**Published:** 2020-10-15

**Authors:** Wilnard Y. T. Tan, Barnaby E. Young, David Chien Lye, Daniel E. K. Chew, Rinkoo Dalan

**Affiliations:** 1 National Centre for Infectious Diseases, Tan Tock Seng Hospital, Lee Kong Chian School of Medicine, Yong Loo Lin School of Medicine, 16 Jalan Tan Tock Seng, Singapore, 308442 Singapore; 2 Department of Endocrinology, Tan Tock Seng Hospital, Lee Kong Chian School of Medicine, 11 Jalan Tan Tock Seng, Singapore, 308433 Singapore

**Keywords:** Risk factors, Endocrine system and metabolic diseases

## Abstract

We aim to study the association of hyperlipidemia and statin use with COVID-19 severity. We analysed a retrospective cohort of 717 patients admitted to a tertiary centre in Singapore for COVID-19 infection. Clinical outcomes of interest were oxygen saturation ≤ 94% requiring supplemental oxygen, intensive-care unit (ICU) admission, invasive mechanical-ventilation and death. Patients on long term dyslipidaemia medications (statins, fibrates or ezetimibe) were considered to have dyslipidaemia. Logistic regression models were used to study the association between dyslipidaemia and clinical outcomes adjusted for age, gender and ethnicity. Statin treatment effect was determined, in a nested case–control design, through logistic treatment models with 1:3 propensity matching for age, gender and ethnicity. All statistical tests were two-sided, and statistical significance was taken as *p* < 0.05. One hundred fifty-six (21.8%) patients had dyslipidaemia and 97% of these were on statins. Logistic treatment models showed a lower chance of ICU admission for statin users when compared to non-statin users (ATET: Coeff (risk difference): − 0.12 (− 0.23, − 0.01); *p* = 0.028). There were no other significant differences in other outcomes. Statin use was independently associated with lower ICU admission. This supports current practice to continue prescription of statins in COVID-19 patients.

## Introduction

The COVID-19 pandemic continues to grow around the world, with more than 20 million cases worldwide^1^. The coronavirus infections, COVID-19, SARS and MERS, are all associated with dysregulated immune and inflammatory processes. Severe cases of COVID-19 are characterised by high circulating pro-inflammatory cytokines concentrations, as well as high neutrophil counts and lymphopenia^2–4^. COVID-19 has been associated with hyperinflammatory states, cardiovascular disease and venous thromboembolism^5–7^.

Inflammation has a potential role in the pathogenesis of dyslipidaemia^8^. Statins are commonly used to treat hyperlipidemia and its pleiotropic effects have been shown to reduce cytokines in various non-infective conditions^9,10^. Long term statin therapy correlates with better outcome in the setting of bacterial pneumonia^11,12^ and influenza^13^. A randomised controlled trial evaluating atorvastatin as a treatment for influenza showed significantly lower levels of inflammatory cytokines with treatment [NCT02056340].

Medical comorbidities such as diabetes, hypertension and cardiovascular diseases have been identified as risk factors for severe COVID-19 in numerous large case series from China, Italy and the United States^14–16^. Dyslipidaemia has not been identified as an independent risk factor^17^, although it is associated with diabetes and hypertension, and contributes to cardiovascular diseases. We aimed to study the association of dyslipidaemia with COVID-19 associated inflammation and the correlation between long term statin therapy and disease severity.

## Methods

We carried out a retrospective cohort study of patients with confirmed COVID-19 hospitalized at the National Centre of Infectious Diseases (NCID), Singapore. NCID is responsible for managing more than 60% of COVID-19 patients admitted to hospital in Singapore. Patients were identified by primary care and emergency doctors based on case definitions informed by evolving epidemiological risk factors, case detection from active contact tracing, enhanced pneumonia surveillance and diagnostic testing based on doctors’ discretion^18^. Patients were included if they were hospitalized from 22 January 2020 to 15 April 2020. This cohort was previously studied for associations with diabetes and hypertension pharmacotherapy^19^ but this analysis is restricted towards studying the association of dyslipidaemia with COVID-19 associated inflammation and the effects of the use of statin with disease severity.

Electronic medical records of hospitalised patients with COVID-19 confirmed by PCR performed on respiratory samples were reviewed to extract information on demographic data on age, gender and ethnicity, presence of comorbidities and concomitant medications, laboratory investigations including full blood count, renal and liver function tests, C-reactive protein (CRP) and lactate dehydrogenase (LDH) and clinical outcomes of COVID-19. Clinical outcomes of interest were hypoxia with oxygen saturation ≤ 94% requiring supplemental oxygen, intensive care unit (ICU) admission and invasive mechanical ventilation (IMV) and death. All study procedures and data collections were performed in accordance with institutional guidelines. The study protocol was reviewed and approved by the Singapore, Ministry of health who provided a waiver of informed consent from study participants for data collection under the Infectious Disease Act as part of national public health research.

Continuous and categorical variables are presented as median (interquartile range) and frequency (%), respectively. We used linear regression models to assess the association between each of the complete blood count variables and inflammatory markers with dyslipidaemia status adjusting for age, gender and ethnicity. To assess the possible treatment effect of statin use on these outcomes we used a nested case–control design, wherein after excluding patients with diabetes and hypertension, we estimated the statin treatment effect through logistic treatment models with 1:3 propensity matching for age, gender and ethnicity. All statistical tests were two-sided, and statistical significance was taken as *p* < 0.05. All statistical analyses were performed using Stata version 15.

## Results

Within our cohort of 717 patients, one hundred fifty-six (21.8%) patients had dyslipidaemia. Individuals with dyslipidaemia were older (62.5 years, IQR 55–68 years versus 37 years, IQR 27–52 years) and more likely to be of Malay ethnicity (18.6% versus 8.9%). Approximately 24–59% of patients had coexisting diabetes, hypertension and atherosclerotic cardiovascular disease defined as history of ischemic heart disease, stroke or peripheral vascular disease. In terms of inflammatory markers, those with dyslipidaemia were more likely to have higher CRP, LDH, procalcitonin, white cell count and neutrophil count but lower lymphocyte count. Patients with dyslipidaemia were more likely to require supplemental oxygen, ICU admission and IMV. The risk of death was higher (*p* < 0.05). See Tables 1 and 2.

**Table 1:** Baseline characteristics and clinical outcomes of patients.

	All	Hyperlipidemia	No Hyperlipidemia
Total number (%)	717 (100)	156 (21.8)	561 (78.2)
Males (%)	410 (57.2)	91 (58.3)	319 (56.9)
Age, median (IQR)	46 (19–57)	62.5 (55–68)	37 (27–52)
Chinese (%)	401 (55.9)	87 (55.77)	314 (55.97)
Malays (%)	79 (11.02)	29 (18.59)	50 (8.91)
Indians (%)	83 (11.58)	22 (14.10)	61 (10.87)
Others (%)	154 (21.48)	18 (11.54)	136 (24.24)
Diabetes (%)	76 (10.60)	58 (37.18)	18 (3.21)
Hypertension (%)	139 (19.39)	92 (58.97)	47 (8.38)
Cardiovascular diseases^a^ (%)	50 (6.97)	38 (24.36)	12 (2.14)
Renal Failure (%)	6 (0.84)	5 (3.21)	1 (0.18)
Systolic BP (mmHg, IQR)	132 (120–143)	139 (132–153)	130 (119–140)
Diastolic BP (mmHg, IQR)	79 (70–87)	78.5 (71.5–88)	79 (70–87)
**Presenting Inflammatory markers and peripheral blood indices**			
CRP, median (mg/L, IQR)	5.3 (1.6–15.9)	12.8 (3.1–47.4)	4.1 (1.3–11.8)
LDH, median (U/L, IQR)	400 (342–500)	473 (377–610)	386 (334–474)
Procalcitonin, median (ug/L, IQR)	0.06 (0.04–0.11)	0.08 (0.04–0.19)	0.05 (0.04–0.08)
White cell count (× 10^9^/L, IQR)	4.9 (4–6.1)	5.3 (4.2–6.7)	4.8 (3.9–5.9)
Neutrophils (× 10^9^/L, IQR)	2.9 (2.11–3.8)	3.3 (2.5–4.4)	2.7 (2.2–3.7)
Platelets (× 10^9^/L, IQR)	204.5 (172–242)	208.5 (165.5–242)	204 (173–242)
Lymphocytes (× 10^9^/L, IQR)	1.3 (1.0–1.7)	1.2 (0.9–1.6)	1.3 (1.0–0.8)
Monocytes (× 10^9^/L, IQR)	0.52 (0.39–0.70)	0.6 (0.4–0.7)	0.51 (0.39–0.70)
**Clinical outcome parameters**			
Supplementary O2 (%)	91 (12.7)	47 (30.1)	44 (7.8)
ICU admission (%)	47 (6.6)	24 (15.4)	23 (4.1)
Intubation (%)	25 (3.5)	14 (9.0)	11 (2.0)
Death (%)	12 (1.67)	7 (4.5)	5 (0.9)
**Use of Concomitant Medications**			
**Anti-hypertensives: n(%)**			
ACE-Inhibitors	28 (3.91)	25 (16.03)	3 (0.53)
Angiotensin-receptor blockers	62 (8.65)	44 (28.21)	18 (3.21)
Beta-blockers	36 (5.02)	21 (13.46)	15 (2.67)
Calcium-channel blockers	68 (9.48)	43 (27.56)	25 (4.46)
Diuretics	18 (2.51)	14 (8.97)	4 (0.71)
**Diabetes Medications**			
Metformin	67 (9.34)	53 (33.97)	14 (2.50)
DPP-4 inhibitors	27 (3.77)	22 (14.10)	5 (0.89)
SGLT-2 inhibitors	16 (2.23)	14 (8.97)	2 (0.36)
Sulfonylureas	33 (4.60)	27 (17.31)	6 (1.07)
Acarbose	3 (0.42)	2 (1.28)	1 (0.18)
Insulin	7 (0.98)	6 (3.85)	1 (0.18)
**Dyslipidaemia Medications**			
Statins	151 (21.06)	151 (96.79)	–
Fibrates	12 (1.67)	12 (7.69)	–
Ezetimibe	10 (1.39)	10 (6.41)	–-

^a^Ischemic heart disease, cerebrovascular accidents, peripheral vascular disease.

**Table 2:** Associations of laboratory markers with hyperlipidemia.

	P value^b^	Coefficient^a^	P value^a^
CRP	< 0.0001*	5.8 (− 2.1 to 13.8)	0.151
LDH	< 0.0001*	5.7 (− 32.0 to 43.3)	0.769
Procalcitonin	0.0069*	− 0.03 (− 0.8 to 0.8)	0.931
White cell count	0.0009*	0.62 (0.2–1.1)	0.005*
Neutrophil count	< 0.0001*	0.64 (0.3–1.0)	0.001*
Haemoglobin	0.0364*	0.21 (− 0.1 to 0.5)	0.115
Platelet count	0.8858	7.26 (− 8.0 to 22.5)	0.350
Lymphocyte count	0.0024*	− 0.01 (− 0.15 to 0.12)	0.849
Monocyte count	0.5151	− 0.02 (− 0.07 to 0.03)	0.535
Haematocrit	0.0110*	− 2.0 (− 5.6 to 1.6)	0.282

^a^Linear regression adjusted for age, gender and ethnicity.

^b^Wilcoxan Rank Sum Test.

*p < 0.05.

Dyslipidaemia was associated with higher white cell count and neutrophil count but not the other inflammatory markers. See Tables 1 and 2.

Of those who had hyperlipidemia, 151 (96.7%) were on statins, 12 (7.7%) were on fibrates and 10 (6.4%) were on ezetimibe. In the nested case–control analysis after excluding patients with diabetes and hypertension, 40 patients were on statins and 509 were non-statin users. The baseline characteristics of these 40 patients is described in Table 3. Approximately 22.5% (9/40) patients had baseline atherosclerotic cardiovascular disease. None of the patients had renal disease or other comorbid diseases. Logistic treatment models using propensity matching showed a lower chance of ICU admission for statin users when compared to non-statin users (Average treatment effect of statins (ATET) Coeff (risk difference): − 0.12 (− 0.23, − 0.01); *p* = 0.028). There were no other significant differences in other outcomes (Table 4).

**Table 3:** Baseline characteristics of patients on statins without comorbid conditions of diabetes or hypertension included in the nested case–control propensity matching analysis.

Variables	Statin users
Total number (%)	40 (100)
Males (%)	22 (55)
Age, median (IQR)	59 (53.5–64)
Chinese (%)	26 (65)
Malays (%)	3 (7.5)
Indians (%)	4 (10)
Others (%)	7 (17.5)
Cardiovascular Disease^a^ (%)	9 (22.5)
Renal Failure (%)	0 (0)
Systolic BP (mmHg, IQR)	138 (130–149)
Diastolic BP (mmHg, IQR)	82 (73–88)
CRP, median (mg/L, IQR)	4.8 (1.9–20.6)
LDH, median (U/L, IQR)	410 (368–536)
Procalcitonin, median (ug/L, IQR)	0.05 (0.04–0.14)
White cell count (× 10^9^/L, IQR)	4.9 (4.0–6.9)
Neutrophils (× 10^9^/L, IQR)	3.15 (2.31–4.19)
Platelets (× 10^9^/L, IQR)	201 (164–228)
Lymphocytes (× 10^9^/L, IQR)	1.13 (0.93–1.51)
Monocytes (× 10^9^/L, IQR)	0.59 (0.46–0.71)
Supplementary O2 (%)	7 (17.5)
ICU admission (%)	1 (2.5)
Intubation (%)	1 (2.5)
Death (%)	2 (5)

^a^Ischemic heart disease, cerebrovascular accidents, peripheral vascular disease.

**Table 4:** Logistic treatment models with 1:3 propensity matching (age, gender, ethnicity) to assess statin treatment effect on clinical outcomes.

	ATET Coeff (95% CI)	*P* value
Hypoxia	− 0.06 (− 0.21, 0.09)	0.449
ICU admission	− 0.12 (− 0.23, − 0.01)	0.028*
Intubation	− 0.08 (− 0.19, 0.02)	0.114
Death	− 0.04 (− 0.16, 0.08)	0.488

*P value < 0.05, ATET: Average treatment effect on statin.

## Discussion

We found that statins was associated with better outcomes in COVID-19. Similar results have been reported from a large study from Hubei province, China wherein they found that statin use was associated with a lower risk of mortality in COVID-19 infections^20^. In another retrospective study, atorvastatin also associated with a lower risk of death in COVID-19 patients admitted to the intensive care unit^21^. In a study involving nursing home residents, statin use was associated with higher chances of asymptomatic infection^22^.

Lipids and cholesterol-rich membrane microdomains facilitates the interaction between the surface glycoprotein S of SARS-CoV and the cellular receptor angiotensin-converting enzyme 2 (ACE2)^23^. Cholesterol has been implicated to have a possible role in the increased risk of infection in the elderly patients wherein higher tissue cholesterol has been shown to increase the endocytic entry of SARS-CoV-2 along with increased trafficking of angiotensin converting enzyme-2 (ACE-2) in a preprint^24^. After cellular entry, RNA viruses require intracellular cholesterol and fatty acids for further replication. For e.g. it has been demonstrated that during the initial phase of dengue virus infection, there is an increase in intracellular cholesterol concentration. This is associated with an increase in low density lipoprotein (LDL) concentrations in cells and a concomitant increase in the enzymatic activity of 3-hydroxy-3-methyl-glutaryl-CoA (HMG-CoA) reductase inside the cells^25,26^. Three decades ago, Mabuchi et al., reported that statins can effectively reduce LDL concentrations through HMG-CoA reductase inhibition^27^ and in the last three decades statins have become the most widely prescribed lipid lowering medication. In COVID-19, statins may help to reduce viral entry and viral transmission by inhibition of the HMG-CoA reductase in the cells which will make less cholesterol available inside cells and tissues.

In our small observational cohort, we observed a significant trend towards higher white cell counts and neutrophil counts in patients with dyslipidaemia. A key pathological process that leads to cardiovascular disease is inflammation. Statins have been shown to have significant pleiotropic, anti-inflammatory and immunomodulatory effects^28–36^, independent of its ability to reduce low-density lipoprotein^36^. Even in rheumatological disease statins are known to modulate the inflammatory response^37^. Additional to its beneficial effects in cardiovascular disease, statins may be beneficial in patients with bacterial sepsis^38,39^, community acquired pneumonia^40^ and influenza^13^. Severe outcomes in COVID-19 is associated with higher markers of inflammation and a “cytokine storm”^41–43^. Statins have the potential to block the molecular mechanisms, including NF-κB and NLRP3 inflammasomes and TLR signalling which are responsible for the "cytokine storm" in severe COVID-19 patients^44–46^.

COVID-19 has been associated with significant cardiovascular complications due to direct effects of SARs-CoV-2 virus with significant effects of the virus on the expression and function of ACE-2 in the vasculature and evidence of coronary endothelial dysfunction and endothelialitis seen in multiple vascular beds in fatal patients with COVID-19^47,48^. Cases of acute coronary events (acute myocardial infarction and thromboembolism) triggered in patients with no underlying history of ischemic heart disease have been reported worldwide^49–51^. Statins are known to be effective in the prevention of endothelial dysfunction and downstream, atherosclerotic pathways and to prevent coronary artery disease^52^. It has been suggested that dyslipidaemia patients maybe at higher risk of atherosclerotic events after recovery from COVID-19. Similar exacerbations have been reported in influenza infections. It has been suggested that statins should be intensified to reduce cardiovascular risk post COVID-19 infection^53,54^.

Hence, we suggest that statins should be continued in dyslipidaemia patients who develop COVID-19 infection . This is especially important in COVID-19 as severe disease is related to cardiovascular comorbidities during infection and increased cardiovascular risk post recovery.

Our study has several limitations. We did not have the quantitative lipid profile on admission to study detailed correlations with the lipid phenotype. We did not have the values of the previous lipid profile including maximum LDL-Cholesterol, so we could not study correlations of cholesterol load with COVID-19 severity. Our cohort was small and adverse outcome rates, were low. This will affect the generalisability of our findings. Lastly, there was a risk of channelling bias as patients on statins were more likely to have more severe cardiovascular disease than those without.

## Conclusion

We found dyslipidaemia patients had a significant trend towards a higher innate immune response shown by higher white cell counts and neutrophil counts. Statin use was independently associated with lower requirement for ICU admission. This supports current practice to continue prescription of statins in hyperlipidemia and other metabolic disorders in COVID-19 patients. The effect of statin use and intensification on COVID-19 disease severity and atherosclerotic events after recovery needs to be studied in larger observational studies or ideally in randomised controlled trials.

## Data Availability

The datasets are available from the corresponding author upon reasonable request.
